# Transgenerational epigenetic inheritance increases trait variation but is not adaptive

**DOI:** 10.1093/evolut/qpaf050

**Published:** 2025-03-11

**Authors:** René S Shahmohamadloo, John M Fryxell, Seth M Rudman

**Affiliations:** School of Biological Sciences, Washington State University, Vancouver, WA, United States; Department of Integrative Biology, University of Guelph, Guelph, ON, Canada; Department of Biology, University of Victoria, Victoria, BC, Canada; School of Biological Sciences, Washington State University, Vancouver, WA, United States

**Keywords:** epigenetics, *Daphnia*, genetic variation, global change, maternal effect, phenotypic plasticity

## Abstract

Understanding organismal responses to environmental change is a central goal of biology with profound implications for the conservation of biodiversity. Widespread evidence of epigenetic modifications in response to environmental stress, including those inherited across generations, has led to considerable speculation about their role in organismal responses to environmental change. Yet, the magnitude and fitness consequences of epigenetic marks carried beyond maternal inheritance are largely unknown. Here, we tested how transgenerational epigenetic inheritance (TEI) shapes the phenotypic response of *Daphnia* clones to the environmental stressor *Microcystis*. We split individuals from each of eight genotypes into exposure and control treatments (P0 generation) and tracked the fitness of their descendants to the F3 generation. We found transgenerational epigenetic exposure to *Microcystis* led to reduced survival and growth rates and no consistent effect on offspring production. TEI was associated with increases in trait variance, suggesting the potential for heritable bet hedging driven by TEI. Taken together, our results demonstrate that TEI causes substantial—but not adaptive—trait shifts, suggesting transgenerational adaptive plasticity may be rare.

## Introduction

Anthropogenic global change is projected to drive significant biodiversity losses this century (Urban et al., 2016), highlighting the need to understand the mechanisms and magnitudes of adaptive phenotypic responses to environmental change (Bellard et al., 2012; Lavergne et al., 2010). While organisms do undergo rapid evolutionary adaptation in response to environmental shifts (Hendry & Kinnison, 1999; Hoffmann & Sgrò, 2011; Rudman et al., 2022), environmentally induced phenotypic plasticity represents the most general and impactful mechanism (Ghalambor et al., 2007; Merilä & Hendry, 2014) and allows organisms to adjust phenotypes in response to the conditions they experience (West-Eberhard, 2003). Yet, most mechanisms underlying plastic shifts do not produce heritable change, limiting the long-term fitness benefits of plasticity when environmental fluctuations are common (Fox et al., 2019).

Nongenetic mechanisms of inheritance, broadly classified as “intergenerational” or “transgenerational,” arise through distinct biological pathways (Sengupta et al., 2023) and could play an important role in the transmission of heritable phenotypic changes in response to environmental fluctuations (Bonduriansky & Day, 2009). Intergenerational inheritance involves the transfer of traits from parent to offspring through mechanisms independent of inherited DNA modifications, such as the acquisition of epigenetic marks during in utero development in live birth species and nonepigenetic mechanisms like maternal resource provisioning (Dowen & Ahmed, 2019; Lockwood et al., 2017; Sengupta et al., 2023). Conversely, transgenerational inheritance involves the transmission of epigenetic information across multiple generations that can persist even in the absence of the original environmental stimulus—known as transgenerational epigenetic inheritance (TEI). The most studied underlying mechanisms of TEI are differential patterns of DNA methylation, histone modifications, and the transmission of noncoding RNAs (Jablonka & Raz, 2009; Jirtle & Skinner, 2007; Sengupta et al., 2023). Despite methodological advances leading to a better understanding of inherited epigenetic marks associated with a variety of stressors across taxa (Felsenfeld, 2014; Fitz-James & Cavalli, 2022; Skinner, 2015), the extent to which epigenetic modifications influence organismal phenotypes and population-level responses remains largely unknown (Proulx & Teotónio, 2017; Proulx et al., 2019).

Hypotheses on the fitness consequences of TEI span widely, including suggestions that they are generally beneficial (hereafter “adaptive”) (Bonduriansky & Day, 2009; Jablonka, 2017; Jablonka & Raz, 2009; Kronholm & Collins, 2016; Lachmann & Jablonka, 1996), neutral (hereafter “nonadaptive”) (Burggren, 2016; Guerrero-Bosagna, 2017; Uller, 2019), or generally detrimental (hereafter “maladaptive”) (Lacal & Ventura, 2018; O’Dea et al., 2016). TEI may confer significant fitness benefits in fluctuating environments (Burggren, 2016; Jablonka, 2017), particularly for organisms with shorter generation times (Houri-Zeevi & Rechavi, 2017). This supposition is based on the assumption of an “epigenetic advantage” (Burggren, 2016; Lind & Spagopoulou, 2018; Rando & Verstrepen, 2007), which posits that TEI facilitates differential gene expression and beneficial phenotypic plasticity, enhancing individual fitness in changing conditions. However, alternative hypotheses suggest that TEI could have neutral or negative fitness effects. For instance, in rapidly fluctuating environments TEI could increase phenotype-environment mismatches. Moreover, a lack of stability of epigenetic marks could erode any fitness benefits across generations (Chevin & Hoffmann, 2017; Lacal & Ventura, 2018; Murray et al., 2022; O’Dea et al., 2016). These contrasting perspectives underscore the importance of determining the contexts in which epigenetic modifications are likely to be beneficial or harmful. Regardless of their fitness consequences, epigenetic modifications—such as differential DNA methylation patterns or histone modifications that influence rates of transcription—are likely to impact organismal phenotypes in ways that can either enhance or detract from fitness, depending on the context (Bossdorf et al., 2008; DeWitt & Scheiner, 2004; Fox et al., 2019; Fusco & Minelli, 2010; Pigliucci, 2001; Vogt, 2021). Evaluating whether TEI simply contributes to phenotypic variance or whether the phenotypic changes from TEI consistently increase fitness requires empirical studies extending to the F3 generation or beyond (Jablonka & Raz, 2009; Jirtle & Skinner, 2007; O’Dea et al., 2016; Sengupta et al., 2023) to disentangle inherited epigenetic modifications from parental and environmentally induced noninherited changes. To date, the limited number of studies measuring the phenotypic effects of TEI in the F3 and later generations (Lacal & Ventura, 2018; Yin et al., 2019) makes drawing definitive conclusions about its effects on phenotypes and fitness tenuous.

Another potential outcome of TEI is an overall increase in phenotypic variance, which could arise through mechanisms that are either adaptive or nonadaptive. The fitness consequences of an increase in variance would likely depend on the environmental context. For instance, “heritable bet hedging,” a strategy whereby organisms produce offspring with a wider range of phenotypes, could enhance population growth rates in fluctuating environments, and hence might be favored by selection (O’Dea et al., 2016). This can occur through deterministic maternal effects, where offspring trait values respond predictably to the maternal environment, or through randomizing maternal effects, where offspring trait values are diversified to hedge against unpredictable future conditions (Proulx & Teotónio, 2017). In contrast, cumulative stress may increase phenotypic variance through the production of offspring with substantial phenotype-environment mismatches; such increases in variance are typically interpreted as reflecting negative consequences for fitness, as they often arise from stress-induced physiological processes (Crain et al., 2008). Measuring TEI effects on fitness-associated phenotypes, both on means and variances, and projecting impacts (Evans et al., 2014) on population dynamics is critical for identifying adaptive or maladaptive phenotypic responses and their potential impact on population persistence in fluctuating environments. Doing so requires documenting TEI effects on fitness, and translating any putative effects to population-level outcomes requires measuring a suite of phenotypes associated with “vital rates” that are the basis for population projection models (Bruijning et al., 2018).

*Daphnia* (water fleas) are a useful model system to test hypotheses about TEI because they reproduce clonally, which facilitates experiments in which genotypes are largely constant across generations (i.e., very little standing genetic variation within clonal lines for evolution to occur) (Harris et al., 2012; Jablonka, 2017; Vogt, 2021). *Daphnia* exhibits visible and measurable phenotypic responses to environmental perturbations that are key to population-level responses, including alterations in morphology, survival, and reproductive strategies (Boersma et al., 1998). Harmful algal blooms (HABs) of the cyanobacterium *Microcystis* are a prominent aquatic contaminant (Harke et al., 2016) that can have both lethal and sublethal effects on a wide range of taxa (Shahmohamadloo et al., 2021, 2023a,b), including *Daphnia* (Ger et al., 2016; Shahmohamadloo et al., 2020a,b). Many *Daphnia* populations show considerable intraspecific genetic variation and evidence of adaptation to HABs (Hairston et al., 2001; Ger et al., 2016; Isanta-Navarro et al., 2021; Shahmohamadloo et al., 2024; Shahmohamadloo et al., 2025). Given the frequent and predictable nature of HABs, *Daphnia*’s tolerance to this stressor aligns with scenarios under which adaptive TEI would be expected to evolve (Bonduriansky & Day, 2009; Chevin & Hoffmann, 2017; Harrisson et al., 2014; Jablonka, 2017; Jablonka & Raz, 2009; Kronholm & Collins, 2016). Studies on *Daphnia* in response to *Microcystis* have documented intergenerational plasticity after one generation of exposure (Asselman et al., 2015, 2017; Gillis & Walsh, 2019; Walsh & Gillis, 2021) and TEI of environmentally induced DNA methylation (Feiner et al., 2022). Testing whether TEI induces phenotypic shifts that impact *Daphnia* fitness in response to HABs provides empirical insight into the adaptive, nonadaptive, or maladaptive role of epigenetic inheritance (O’Dea et al., 2016) in a model system that has considerable ecological and conservation importance as a potential remediator of HABs (Sarnelle & Wilson, 2005).

**Figure 1.**
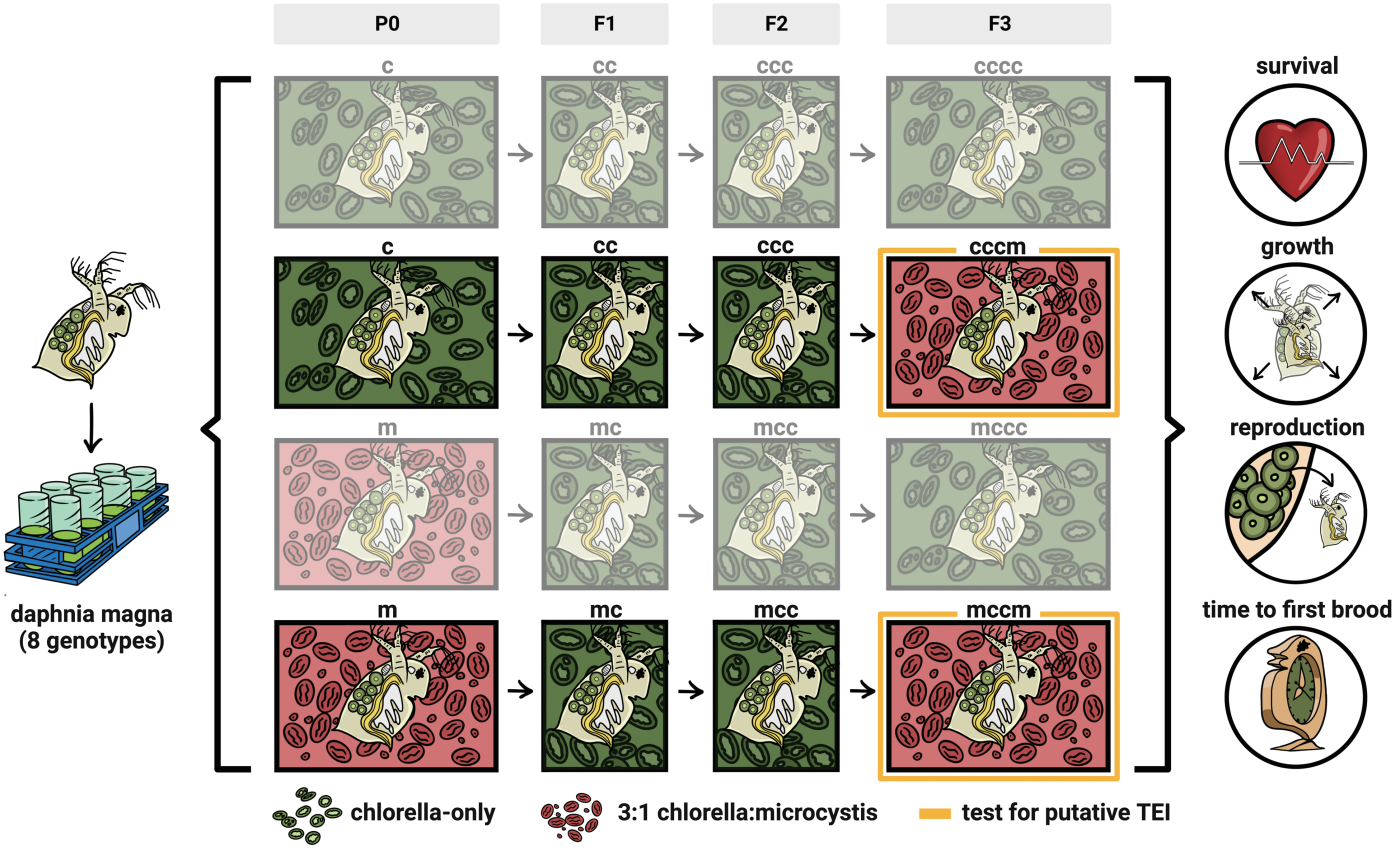
A schematic diagram illustrates the experimental design testing transgenerational epigenetic inheritance (TEI) in *Daphnia magna*. Eight genotypes were initially reared on either a Chlorella-only diet (denoted by ‘c’) or a 3:1 Chlorella:Microcystis diet (denoted by ‘m’) in the P0 generation. Offspring (F1 and F2) were maintained on Chlorella-only. In the F3 generation, individuals were split into two treatments: Chlorella-only (cccm) or re-exposure to Microcystis (mccm, test for putative TEI). Fitness traits such as survival, growth, reproduction, and time to first brood were measured. Icons representing these fitness traits appear to the right of the diagram.

To determine the phenotypic and demographic consequences of TEI, we empirically investigated the following questions: (1) Does TEI influence mean phenotypes? (2) If TEI influences mean phenotypes, is the direction of phenotypic change primarily adaptive, nonadaptive, or maladaptive? And, (3) Does TEI influence the amount of phenotypic variation? To determine whether TEI influences fitness-associated traits and population-level dynamics in *Daphnia* we compared two F3 exposure groups: “cccm” (i.e., no great-grandmaternal exposure to *Microcystis* in P0 followed by great-granddaughter exposure to *Microcystis* in F3) and “mccm” (i.e., great-grandmaternal exposure to *Microcystis* in P0 followed by great-granddaughter exposure to *Microcystis* in F3) repeated across 8 unique *Daphnia* clones (Figure 1). We additionally measured TEI by assessing the effect of P0 exposure through a comparison of reaction norms, where P0 and F3 environments differed consistently across P0 exposure contrasts (“cccc” - “cccm” vs “mccc” - “mccm”). We quantified the chronic effects of the toxigenic cyanobacterium *Microcystis* on their life-history traits (survival, body growth, number of neonates produced, eye size, and maturation rate) in the final generation (F3). Clonal replication allows for an assessment of the effects of TEI averaged across multiple genetic backgrounds. By employing fitness-associated phenotypes measured across these eight distinct tests within a simple population matrix model, we test for the effects of TEI on vital rates. This direct investigation provides valuable insights into a potentially significant mechanism governing organismal responses to environmental change, while also addressing critical questions (Jablonka, 2017; O’Dea et al., 2016) regarding the adaptive, nonadaptive, or maladaptive nature of TEI’s influence on mean phenotypes and its impact on the variance of phenotypic traits.

## Methods

### *Daphnia magna* field collection and culturing

Eight genotypes of *D. magna* were collected from “Langerodevijver” (LRV; 50° 49′ 42.08″, 04° 38′ 20.60″), a large waterbody (surface area = 140,000 m^2^, max depth = 1 m) within the nature reserve of Doode Bemde, Vlaams-Brabant, Belgium (Orsini et al., 2012). In previous work whole genome sequences of these clones showed they are genetically distinct and that tolerance to cyanobacteria is not correlated with metrics of genomic-wide divergence between them (Shahmohamadloo et al., 2023c). Like many temperate freshwater ecosystems LRV has yearly seasonal *Microcystis* HABs and contains a large resident population of *D. magna* (Luc de Meester *person. comm.*). Parthenogenetic lines of each genotype were maintained for over five years in continuous cultures in UV-filtered dechlorinated municipal tap water containing 2 mg C L^−1^ of the green alga *Chlorella vulgaris* (strain CPCC 90; Canadian Phycological Culture Centre, Waterloo, ON, Canada). *C. vulgaris* was grown in COMBO medium (Kilham et al., 1998).

### *Microcystis aeruginosa* culturing

Following a previously described method (Shahmohamadloo et al., 2019), *M. aeruginosa* (strain CPCC 300; Canadian Phycological Culture Centre, Waterloo, ON, Canada) was cultured in BG-11 media and kept in a growth chamber under axenic conditions with a fixed temperature of 21 ± 1 °C, cool-white fluorescent light of 600 ± 15 lx, with a photoperiod of 16:8 h light:dark. The culture was grown for a minimum of one month before preparation for the transgenerational plasticity study. *M. aeruginosa* CPCC 300 produces microcystins-LR (CAS: 101043-37-2, C_49_H_74_N_10_O_12_) and its desmethylated form [D-Asp³]-microcystin-LR (CAS: 120011-66-7, C_48_H_72_N_10_O_12_), which occur widely in freshwater ecosystems (Chorus & Welker, 2021; Harke et al., 2016) and are toxic to many zooplankton species.

To prepare *M. aeruginosa* for testing on *D. magna*, an aliquot of the stock was inoculated in 100% COMBO medium for two weeks prior to test initiation and cultured to a cell concentration of 1.2 ± 0.02 × 10^7^ cells mL^−1^. This medium was chosen because it supports the growth of algae and cyanobacteria and is nontoxic to zooplankton (Kilham et al., 1998).

### Transgenerational study

We evaluated within- and across-generation responses to *M. aeruginosa* using eight genotypes of *D. magna*. Phenotypic responses measured include survival, body growth, reproduction (number of offspring produced), eye size, and maturation rate (calculated as the inverse of the time to first brood).

To prepare for this study, we isolated one adult female *D. magna* per genotype in separate 50-mL glass tubes inoculated with COMBO medium and *C. vulgaris* at 2 mg C L^−1^, and monitored them daily for reproduction. For each genotype, 10 individuals (80 individuals total) were established as *founding mothers* to generate offspring for the transgenerational study (P0 generation). These founding mothers were incubated under constant conditions (temperature of 21 ± 1 °C, cool-white fluorescent light of 600 ± 15 lx, with a photoperiod of 16:8 hr light:dark).

To run this study, we reared P0 *D. magna* in one of two common gardens: Chlorella-only (optimal diet) and 3:1 Chlorella:Microcystis (toxic diet). Both common gardens provided *D. magna* with 2 mg C L^−1^, corresponding to 3 × 10^6^ cells total and corroborates with previous literature exposing daphnids to dietary combinations of green algae and cyanobacteria (Isanta-Navarro et al., 2021; Rohrlack et al., 2005; Shahmohamadloo et al., 2020a). The 3:1 Chlorella:Microcystis treatment was additionally chosen because these ratios exist in the wild (Chorus & Welker, 2021; Harke et al., 2016) and can cause sublethal, intergenerational effects in *D. magna* (Shahmohamadloo et al., 2020a,b).

For the experimental phase, a minimum of 40 replicates per P0 *D. magna* genotype (offspring produced by the founding mothers) were individually raised in 50-mL tubes and fed their respective diets 3 × per week until they produced their first broods. All offspring across treatments were then reared for two generations—F1 and F2—in Chlorella-only until they too produced their first broods. The F2 offspring were then split in half for the F3 generation. The first subset of individuals (>20) from each clone were exposed to Chlorella-only until their first brood was produced. The second subset of individuals (>20) were exposed to 3:1 Chlorella:Microcystis. This combination of treatments generated a minimum of 40 clonal replicates per original *D. magna* genotype in generation P0, ensuring minimal within-generation genetic variation among individuals of the same genotype. Previous work showed the magnitude of intraspecific genetic variation in the survival, growth, reproduction, and time to first brood of clones was significantly influenced by the presence of *M. aeruginosa* (Shahmohamadloo et al., 2024; Shahmohamadloo et al., 2025). To ensure 40 replicates per F2 *D. magna* genotype would survive to the final generation of F3 before it was split in half, we maintained additional replicates for certain genotypes that were particularly sensitive to *M. aeruginosa* toxicity. We individually monitored and recorded the lineage of each *D. magna* replicate, tracing maternal relationships from each mother to its daughter (P_x_ to P_x+1_) and from each great-grandmother to its great-granddaughter (P0 to F3) across all genotypes and common garden conditions. In summary, the experiment required a minimum of 640 P0 *D. magna* and 2,560 *D. magna* raised across all four generations, spanning 100 days (Figure 1).

Since this was a semistatic test (i.e., the medium was periodically renewed to maintain water quality and nutrient levels), solutions were renewed 3 × wk by transferring *D. magna* from old to new glass tubes, followed by supplying each *D. magna* with 3 × 10^6^ cells of food, corresponding with 2 mg C L^−1^. All *D. magna* were transferred at consistent intervals (3 × wk) to ensure uniform handling and standardized algal and water quality conditions. This approach followed standardized toxicity protocols for testing of *D. magna* (OECD, 2012) and minimized variability in algal availability and toxicity within treatments. Survival, reproduction, and the timing of first brood were recorded daily. Growth and eye size (mm) for each replicate across genotypes and common gardens were also measured on days 0, 3, 7, and day of the first brood for P0 and F3 to assess for TEI impacts within and across genotypes and treatment effects. The study was incubated under 400–800 lx cool-white fluorescent light at 20 ± 1 °C with a 16:8 light:dark cycle. Water chemistry parameters were measured at initiation, solution changes, and termination of the test.

### Statistical analysis

We tested for the effects of TEI using generalized linear mixed models (GLMM) for each phenotypic response in which “P0 exposure” and “F3 exposure exposure” were treated as fixed effects and ‘*Daphnia* clone’ was treated as a random effect. We used these models to determine whether TEI had significant effects on phenotypes within two distinct planned contrasts. First, we tested whether great-grandparental exposure to *M. aeruginosa* (i.e., “mccm” vs. “cccm”) resulted in significant phenotypic differences. Second, we compared the effects of TEI on reaction norms through a planned contrast: “cccc” - “cccm” (environmental effects only) and “mccc” - “mccm” (environmental + TEI effects). In this comparison, slopes that show a greater negative effect of P0 “m” exposure in the F3 generation would be indicative of a maladaptive trait shift. We fit appropriate link functions for each GLMM. For survival data, we used a binomial distribution and logit link. For neonate production data, we used a linear mixed effects model (LME). For body growth to day 7, and maturation rate datasets, we used a Poisson GLMM and log-link function.

For inferences on population impacts from TEI, Leslie matrices were constructed for survival and reproduction for F3 exposure to “mccm” vs. “cccm.” These matrices included exact age classes based on the time from birth to first brood, providing additional temporal resolution in reproduction rates. The population growth rate (*λ*) and net reproductive rate (*R*_0_) were calculated using the Euler-Lotka equation. *R*_0_ was determined as the exact number of offspring produced by all mothers in the F3 generation for each clone and exposure combination (Caswell, 2000). Because all mothers produced only a single clutch, this approach provides a straightforward estimate of *R*_0_ based on observed offspring production. *λ* was estimated iteratively to satisfy the Euler-Lotka equation, based on early life survival and reproduction data (birth to time of first brood). The difference in neonate production between “mccm” and “cccm” from F3 exposure were calculated for each “clone” and “exposure” combination and plotted. The difference in variance of neonate production between “mccm” and “cccm” from F3 exposure was also calculated for each “clone” and “exposure” combination and plotted. We applied Bartlett’s test to assess the homogeneity of variances in neonate production between the “mccm” and “cccm” F3 treatment groups of *D. magna* mothers. Before performing this test, we verified the normality of neonate production data using the Shapiro–Wilk test (*p* > 0.05), confirming the suitability of Bartlett’s test for this analysis.

For all analyses, the *p*-level significance cutoff was 0.05. All statistical analyses were completed in R version 4.2.2 (R Core Team, 2022). The data supporting the results are archived in the public repository Dryad under http://www.doi.org/10.5061/dryad.ffbg79d31.

## Results

### P0–F2 generation impacts from exposure

All eight *Daphnia* genotypes had 100% survival up to their first reproductive event across all replicates in P0 → F2 when exposed to Chlorella-only (“c” → “ccc”). However, exposure to *Microcystis* (“m”) caused a decrease in survival to an average of 67% across all *Daphnia* genotypes in P0, ranging from 57% survival of “genotype 5” to 84% survival of “genotype 7.” Following cessation of *Microcystis* exposure, subsequent generations returned to near total survival to age at first brood (e.g., F1 (“mc”) had 97% survival and F2 (“mcc”) had 99% survival on average across all *Daphnia* genotypes (see Dryad repository)).

### Great-grandmaternal exposure × F3 generation interactions

**Figure 2.**
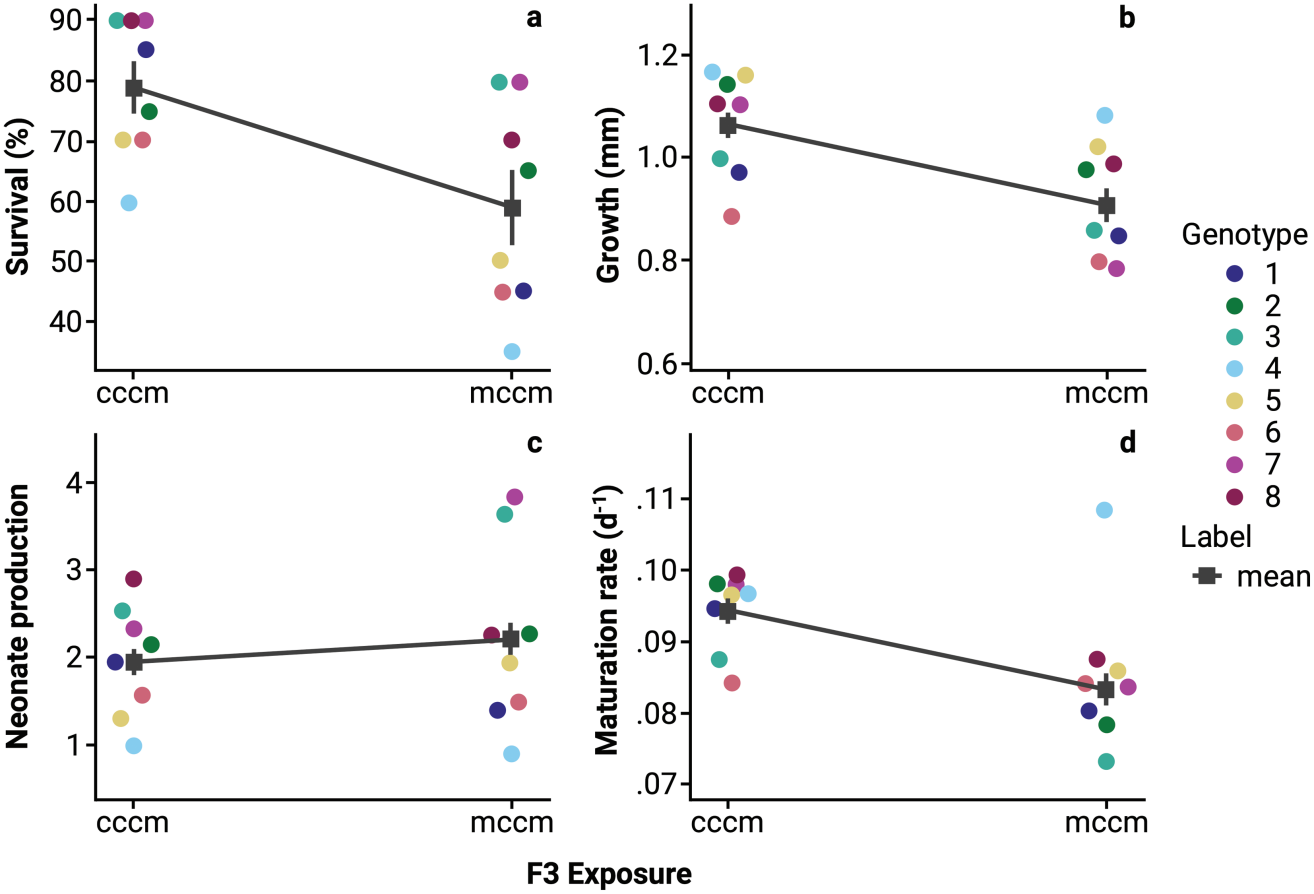
Phenotypic variation in (A) survival at day 7, (B) growth at day 7, (C) neonate production per *Daphnia magna*, and (D) maturation rate across eight *D. magna* clonal populations after four generations (P0 → F3) of transgenerational epigenetic inheritance. The mean (■) ± SE for each phenotype is designated by a line. *D. magna* exposed to *Microcystis aeruginosa* in P0 and F3 are signified by ‘mccm’, and *D. magna* only exposed in F3 are signified by “cccm.”

TEI, as measured on the F3 generation, caused a significant decrease in survival (*t*_(1,504)_ = 3.39 *p* < 0.01 0; Figure 2A); *Daphnia* from “cccm” had survival rates of 78.75 ± 4.09% over 7 days compared to *Daphnia* from “mccm” who had survival rates of 58.75 ± 6.11% survival over 7 days. Similarly, we observed a significant decrease in body growth (*t*_(1,505)_ = 4.17, *p* < 0.01; Figure 2B); *Daphnia* from “cccm” grew 1.06 ± 0.02 mm over 7 days compared to *Daphnia* from “mccm” which grew 0.91 ± 0.03 mm. We further observed a significant delay in maturation rate (*t*_(1,503)_ = 5.79, *p* < 0.01; Figure 2D); *Daphnia* from “cccm” reproduced at a rate of 0.094 ± 0.0015 d^−1^ compared to *Daphnia* from “mccm” which reproduced at a rate of 0.083 ± 0.0018 d^−1^. We did not observe significant effects of TEI on neonate production per *Daphnia* (*t*_(1,505)_ = −1.62, *p* = 0.11; Figure 2C). However, neonate production per surviving *Daphnia* in the F3 generation was greater with TEI (*t*_(1,505)_ = −5.29, *p* < 0.01; “mccm” was 4.14 ± 0.19 and for “cccm” was 2.67 ± 0.10). TEI did not produce detectable changes in eye size (*t*_(1,505)_ = −0.078, *p* = 0.94; Supplementary Figure S1, Supporting Information), contrary to the hypothesis proposing maternal effects on offspring eye size as an adaptive response linked to improved foraging abilities (Walsh & Gillis, 2021).

### Reaction norms for F3 phenotypes

**Figure 3.**
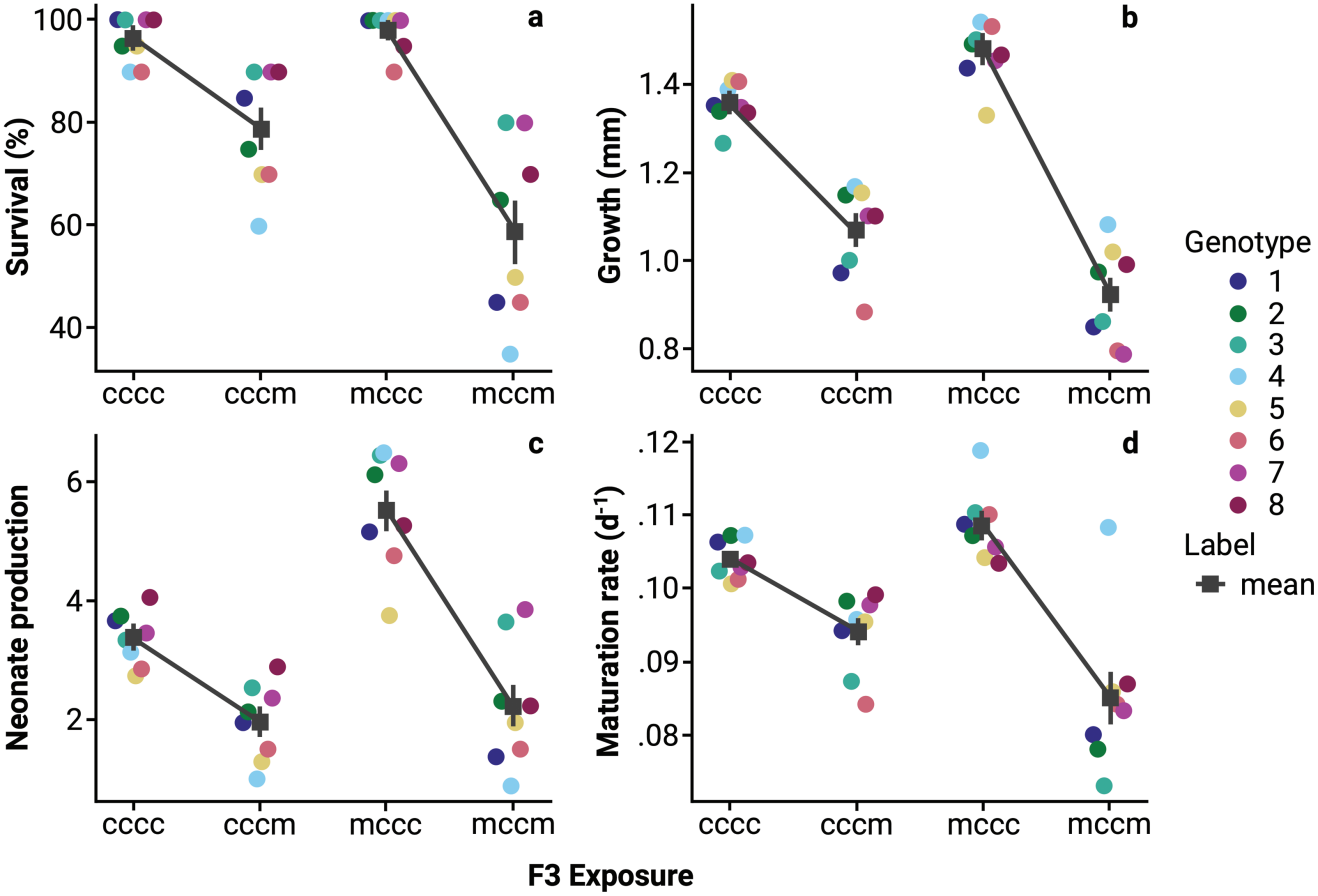
Reaction norms to disentangle the effects of P0 exposure to *Microcystis* by comparing “cccc” - “cccm” (environmental effects only) and “mccc” - “mccm” (environmental + TEI effects) in the F3 generation for (A) survival at day 7, (B) growth at day 7, (C) neonate production per *Daphnia magna*, and (D) maturation rate across eight *D. magna* clonal populations after four generations (P0 → F3) of transgenerational epigenetic inheritance. The mean (■) ± SE for each phenotype is designated by a line. *D. magna* exposed to *Chlorella vulgaris* or *Microcystis aeruginosa* is signified by a “c” or “m,” respectively, sequentially ordered by the generation of exposure.

As an additional test of the effects of TEI on phenotypes we constructed reaction norms to disentangle the effects of P0 exposure to *Microcystis*, with a focus on comparing “cccc” - “cccm” (environmental effects only) and “mccc” - “mccm” (environmental + TEI effects) norms in the F3 generation for each phenotype (Figure 3). In all cases, the overall effect of P0 exposure led to a putative detrimental effect on phenotype relative to no P0 exposure as demonstrated by more strongly negative slopes (Figure 3). Survival showed significant increases in slope differences between “cccc *vs* cccm” and “mccc vs. mccm” conditions across all *Daphnia* genotypes (*t*_(1,504)_ = −2.29, *p* = 0.022, mean difference = 21.88, 95% CI: 12.41–31.34; Figure 3A). Similarly, neonate production exhibited a significant increase in slope differences between conditions (*t*_(1,504)_ = −3.90, *p* < 0.01, mean difference = 1.90, 95% CI: 1.17–2.62; Figure 3C), highlighting the consistent negative effects of *Microcystis* exposure on reproductive output. Maturation rate also showed a significant increase in slope differences between conditions (*t*_(1,502)_ = −6.41, *p* < 0.01 mean difference = 0.0014, 95% CI: 0.0075–0.020; Figure 3D), suggesting that reproductive timing is impacted by TEI effects. Finally, body growth displayed significant reductions under *Microcystis* conditions (*t*_(1,504)_ = −5.69, *p* < 0.01, mean difference = 0.26, 95% CI: 0.17–0.36; Figure 3B), reinforcing the observed maladaptive impacts of great-grandmaternal exposure to *Microcystis* on subsequent generations.

### Great-grandmaternal exposure × population growth impacts

**Figure 4.**
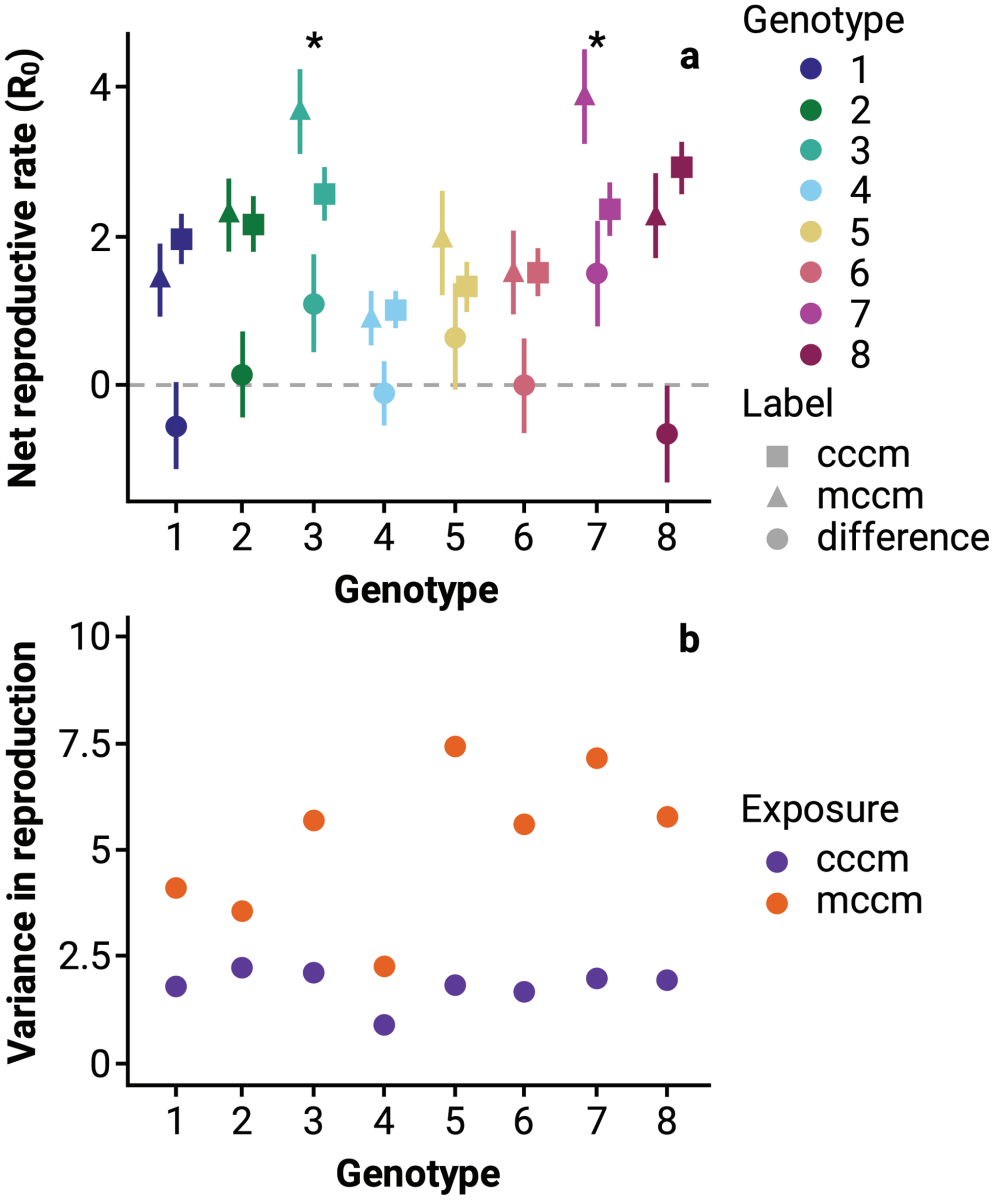
(A) Difference in neonate production (“mccm” – “cccm”) between exposures to *Microcystis aeruginosa* across each of eight *Daphnia magna* clonal populations in the F3 generation. The net reproductive rate (*R*_0_) for “cccm” ± SE (■), “mccm” ± SE (▲), and the net difference between “mccm” and “cccm” ± SE (●) are listed for each genotype. Positive values (>0) indicate a beneficial effect on population dynamics, while negative values (<0) indicate a detrimental effect. Significance between treatments are designated by an asterisk (*) for each genotype. (B) The variance in neonate production of “mccm” and “cccm” exposures to *M. aeruginosa* across eight *D. magna* clones in the F3 generation. See Supplementary Figures S2 and S3 and Supplementary Tables S1 and S2 for similar analyses on growth and maturation rate.

To assess the potential impact of TEI on population growth rates, we constructed Leslie matrices with observed mean rates of survival and neonate production from F3 exposure to “cccm” and “mccm.” We calculated the net reproductive rate (*R*_0_) for each F3 *Daphnia* clonal population, and the mean difference in *R*_0_ between “mccm” and “cccm” for each “clone” and “exposure” combination was measured to determine whether TEI exposure in P0 would be positive (*R*_0_ > 0), null (*R*_0_ = 0), or negative (*R*_0_ < 0) relative to populations with no mechanism for TEI (Figure 4A). The mean difference in *R*_0_ between “mccm” (2.23) and “cccm” (1.96) for all clones was 0.26, indicating that the overall demographic impact on *Daphnia* clones was neutral.

### Variance in neonate production across exposures

Beyond shifts in trait means, changes in the variance of fitness-associated traits can have a profound impact on population persistence by influencing phenotypic diversity (Bolnick et al., 2011; Rodriguez-Cabal et al., 2017). Greater phenotypic diversity enables populations to better withstand environmental change by buffering against extreme conditions or novel stressors. It also provides a broader range of traits on which natural selection can act, facilitating rapid evolutionary responses to selection (Barrett & Schluter, 2008; Rennison et al., 2019). Thus, we next compared the variances of neonate production for the “mccm” and “cccm” treatment groups of *D. magna* mothers from F3 exposure using Bartlett’s test (Figure 4B). *Daphnia* with TEI exposure to *Microcystis* had significantly greater variance than *Daphnia* from “cccm” whose great-grandmothers were not exposed to *Microcystis* (Bartlett’s *K*^2^ = 10.48, *df* = 1, *p* = 0.0012).

## Discussion

### Phenotypic and population-level effects of great-grandparental exposure

The phenotypic effects associated with TEI exposure as measured in the F3 generation were considerable and the mean shifts we observed tended towards a maladaptive response across the 8 unique *Daphnia* genotypes (Figures 2 and 3). When phenotypes were combined into a population projection, TEI did not lead to a difference in the overall population growth rate based on R_0_ (Figure 4A). TEI did increase variance in reproductive output (Figure 4B).

The environmental stressor and concentration utilized for this study were chosen to maximize the chance of seeing TEI. Toxic *Microcystis* is a common stressor in freshwater ecosystems and our concentrations were based on environmental values and prior work cataloging lethal and sublethal toxicity effects (Chislock et al., 2013; Harke et al., 2016; Isanta-Navarro et al., 2021; Shahmohamadloo et al., 2020a). Moreover, prior work has documented notable transgenerational epigenetic modifications via DNA methylation in *Daphnia* exposed to *Microcystis* (Feiner et al., 2022).

### TEI as a generally adaptive mechanism of phenotypic change

The potential for epigenetic mutations to contribute to adaptation has been extensively modeled. Epigenetic mutations, if stable and beneficial, can significantly influence the rate and outcome of adaptation by speeding up the initial stages of “adaptive walks,” a progression wherein successive beneficial mutations drive a population closer to an optimal level of fitness (Kronholm & Collins, 2016). However, the lasting evolutionary impact of these mutations often hinges on subsequent genetic assimilation, whereby epigenetically induced traits become stably encoded in the genome through selection (Kronholm & Collins, 2016). The impact of transgenerational epigenetic mutations on fitness values crucially depends on their stability, phenotypic effect, duration of the effect, and duration of the stressor (Geoghegan & Spencer, 2013; Jablonka & Raz, 2009; Plaistow et al., 2006). For TEI, if epigenetic mutations are unstable or have negative fitness effects, they may not persist across generations or may even hinder adaptive evolution (Kronholm & Collins, 2016). This theory runs contrary to other existing models suggesting the inheritance of acquired epigenetic variations can be adaptive across a wide range of environmental conditions (Rivoire & Leibler, 2014) and can be beneficial in environments marked by predictable fluctuations (Jablonka, 2017). Recent population genetic models incorporating epigenetic variation further demonstrate the potential for stable epialleles to be maintained under neutral conditions and for epialleles compensating for deleterious mutations to deviate from mutation-selection balance, indicating a possible contribution of transient epigenetic regulation to the maintenance of genetic and epigenetic variation in populations (Webster & Phillips, 2024). The latter theories are supported by recent experimental work in clonal yeast populations demonstrating that epigenetic switching, despite its instability, has adaptive advantages under particular fluctuating environments and can persist at low frequencies even in conditions predicted to be detrimental to epigenetic switchers (Stajic et al., 2022).

When translating the conclusions from these models to natural populations and environments the potential for adaptive TEI to contribute to population persistence may depend largely on the periodicity and predictability of environmental fluctuations. TEI could allow populations to better persist in fluctuating environments, but we suggest that such conditions—where TEI would provide a significant fitness benefit—are likely to be rare. Any potential benefits of TEI might be limited to specific scenarios where environmental changes are recurrent and predictable, as opposed to random or erratic stressors. Moreover, the evolution of TEI could depend strongly on any costs associated with epigenetic marks, and associated phenotypic shifts, when stressors are not present. With short generation times and inhabiting environments marked by intense seasonal HABs of *Microcystis*, *Daphnia* fit several key criteria under which the evolution of adaptive TEI could evolve. Moreover, we see intraspecific variation for TEI within genotypes derived from a single population (Figure 4A). However, across individual phenotypes and overall effects on population dynamics we do not see evidence for adaptive TEI. This could stem from several additional possible limitations for the evolution of adaptive TEI. Epigenetic marks, such as DNA methylation or histone modifications, may not persist long enough for selection to effectively act on them due to their instability (Jablonka, 2017; Jablonka & Lamb, 2010). These marks can be reversible and dynamic, potentially erasing or modifying in response to environmental changes or cellular processes (Chen et al., 2016; Fitz-James & Cavalli, 2022; Jablonka, 2017). Given this instability, rapid adaptation from standing genetic variation might ultimately confer greater evolutionary advantages than selection acting on TEI. Cases of rapid adaptation include evolution of phenotypic plasticity and intergenerational epigenetic inheritance, prominent in *Daphnia* responses to *Microcystis* (Hairston et al., 1999, 2001; Isanta-Navarro et al., 2021). Our results support this; across environmental conditions (Chlorella-only and 3:1 Chlorella:Microcystis (present work), but also more severe HAB exposures (2:1 and 1:1 Chlorella:Microcystis (Shahmohamadloo et al., 2023c)), the eight unique genotypes of *Daphnia* show both strong and consistent patterns of variation in fitness-associated phenotypes.

Ultimately, the stability of epialleles, the frequency and predictability of environmental shifts, and the associated costs of epigenetic resetting via TEI, among other factors, may lead TEI to produce complex and unpredictable phenotypic outcomes (Herman et al., 2014; O’Dea et al., 2016). Together with a range of constraints, and other mechanisms by which phenotype-environment mismatches might be reduced, this may limit the frequency of adaptive TEI in nature. Overall, our data support hypotheses that environmentally induced epigenetic changes are transgenerationally inherited, but rarely adaptive (Heard & Martienssen, 2014). Additional empirical work to build a larger number of test cases would be beneficial, though the number of eukaryotic taxa that are readily amenable to the type of experimentation needed may be somewhat limited.

### TEI increases variance—bet hedging or cumulative stress

A notable directional effect of TEI on fitness-associated phenotypes we observed was an increase in phenotypic variation—all 8 *Daphnia* clones had higher variation in F3 reproductive output with prior P0 exposure (Figure 4). This result could reflect two potential mechanisms: (1) an adaptive mechanism, such as heritable bet hedging, where increased variation enhances survival in unpredictable environments, or (2) a maladaptive response to cumulative stress, where increased variation reflects compromised fitness due to environmental pressures. Heritable bet hedging describes cases where increased phenotypic variability provides a hedge against unpredictable environmental changes, increasing the likelihood of population persistence under fluctuating conditions (O’Dea et al., 2016). Cumulative stress suggests repeated stressors could induce transgenerational effects on genome regulation that are maladaptive, potentially shifting the mean phenotype. However, in some cases, cumulative stress could also increase phenotypic variance if individuals respond to stress heterogeneously, for instance, due to differential epigenetic regulation or stochastic effects. Recent observations of compounded epigenetic impacts and disease susceptibility from successive multigenerational exposure to different toxicants in rats (Nilsson et al., 2023) demonstrates epigenetic modifications associated with cumulative stress. This highlights a more nuanced relationship between developmental plasticity, genetic mechanisms, and environmental change in shaping population dynamics.

The way in which increases in phenotypic variance influence the fate of populations dictates how the observed patterns of increased variance in our data should be interpreted. Increases in variation could have important and direct effects on populations, such as those described by Jensen’s inequality (Bolnick et al., 2011), or they could simply be maladaptive and lead to demographic costs. Over longer durations reliance on bet hedging strategies may result in extinction due to directional environmental changes (O’Dea et al., 2016), particularly in cases where stabilizing selection maintains a narrow range of trait values (Chevin & Hoffmann, 2017). More empirical data is needed to understand whether TEI generally increases phenotypic variance. Supplemented by further empirical investigations that measure or model the interaction between increased trait variation and varying amounts—as well as periodicity—of environmental variation, this approach could reveal whether the observed patterns of increased variance resulting from TEI confer adaptive advantages and are potentially significant for the maintenance of biodiversity (Harmon & Pfennig, 2021).

Our study tests an array of competing hypotheses regarding the fitness effects of TEI in response to environmental stress. TEI exposure of *Daphnia* to *Microcystis* in P0 did not yield significant adaptive changes in fitness-associated phenotypes, instead revealing a propensity for maladaptive responses across clones. The absence of discernible effects on population growth rates rejects the hypothesis that TEI enhances population-level responses by *Daphnia* to cyanobacteria exposure. The observed increase in trait variation suggests there may be interesting potential for heritable bet hedging, with higher variance potentially capable of influencing population persistence under challenging conditions. Our study highlights the need for the construction of TEI models that better reflect the nuanced interactions among environmental stress, epigenetic inheritance, and the action of other mechanisms (i.e., phenotypic plasticity, adaptation from standing genetic variation) that may reduce phenotype-environment mismatches. Existing theoretical frameworks often fail to incorporate the complexity of these interactions, particularly under temporally fluctuating environmental conditions. While additional empirical investigations across taxa should be done to continue to elucidate TEI’s role in fitness in fluctuating environments (O’Dea et al., 2016), a reevaluation of its importance is also warranted.

## Supplementary material

Supplementary material is available online at *Evolution*.

qpaf050_suppl_Supplementary_Figures_S1-S3_Tables_S1-S2

## Data Availability

The data supporting the results are archived in the public repository Dryad under DOI http://www.doi.org/10.5061/dryad.ffbg79d31.

## References

[CIT0001] Asselman, J., De Coninck, D. I., Beert, E., Janssen, C. R., Orsini, L., Pfrender, M. E., Decaestecker, E., & De Schamphelaere, K. A. (2017). Bisulfite sequencing with daphnia highlights a role for epigenetics in regulating stress response to microcystis through preferential differential methylation of serine and threonine amino acids. Environmental Science and Technology, 51(2), 924–931. https://doi.org/10.1021/acs.est.6b03870 27983812

[CIT0002] Asselman, J., De Coninck, D. I. M., Vandegehuchte, M. B., Jansen, M., Decaestecker, E., De Meester, L., Vanden Bussche, J., Vanhaecke, L., Janssen, C. R., & De Schamphelaere, K. A. C. (2015). Global cytosine methylation in *Daphnia magna* depends on genotype, environment, and their interaction. Environmental Toxicology and Chemistry, 34, 1056–1061. 25639773 10.1002/etc.2887

[CIT0003] Barrett, R. D. H., & Schluter, D. (2008). Adaptation from standing genetic variation. Trends in Ecology and Evolution, 23, 38–44. 18006185 10.1016/j.tree.2007.09.008

[CIT0004] Bellard, C., Bertelsmeier, C., Leadley, P., Thuiller, W., & Courchamp, F. (2012). Impacts of climate change on the future of biodiversity. Ecology Letters, 15, 365–377. 22257223 10.1111/j.1461-0248.2011.01736.x PMC3880584

[CIT0005] Boersma, M., Spaak, P., & De Meester, L. (1998). Predator-mediated plasticity in morphology, life history, and behavior of *Daphnia*: The uncoupling of responses. American Naturalist, 152(2), 237–248. https://doi.org/10.1086/286164 18811388

[CIT0006] Bolnick, D. I., Amarasekare, P., Araújo, M. S., Bürger, R., Levine, J. M., Novak, M., Rudolf, V. H. W., Schreiber, S. J., Urban, M. C., & Vasseur, D. A. (2011). Why intraspecific trait variation matters in community ecology. Trends in Ecology and Evolution, 26, 183–192. 21367482 10.1016/j.tree.2011.01.009 PMC3088364

[CIT0007] Bonduriansky, R., & Day, T. (2009). Nongenetic inheritance and its evolutionary implications. Annual Review of Ecology, Evolution, and Systematics, 40(1), 103–125. https://doi.org/10.1146/annurev.ecolsys.39.110707.173441

[CIT0008] Bossdorf, O., Richards, C. L., & Pigliucci, M. (2008). Epigenetics for ecologists. Ecology Letters, 11(2), 106–115. https://doi.org/10.1111/j.1461-0248.2007.01130.x 18021243

[CIT0009] Bruijning, M., ten Berge, A. C. M., & Jongejans, E. (2018). Population‐level responses to temperature, density and clonal differences in *Daphnia magna* as revealed by integral projection modelling. Functional Ecology, 32(10), 2407–2422. https://doi.org/10.1111/1365-2435.13192

[CIT0010] Burggren, W. (2016). Epigenetic inheritance and its role in evolutionary biology: Re-evaluation and new perspectives. Biology, 5(2), 24. https://doi.org/10.3390/biology5020024 27231949 PMC4929538

[CIT0011] Caswell, H. (2000). Matrix population models. 1. Sinauer.

[CIT0012] Chen, Q., Yan, W., & Duan, E. (2016). Epigenetic inheritance of acquired traits through sperm RNAs and sperm RNA modifications. Nature Reviews Genetics, 17(12), 733–743. https://doi.org/10.1038/nrg.2016.106 PMC5441558 27694809

[CIT0013] Chevin, L.-M., & Hoffmann, A. A. (2017). Evolution of phenotypic plasticity in extreme environments. Philosophical Transactions of the Royal Society of London. Series B, Biological Sciences, 372(1723), 20160138. https://doi.org/10.1098/rstb.2016.0138 28483868 PMC5434089

[CIT0014] Chislock, M. F., Sarnelle, O., Jernigan, L. M., & Wilson, A. E. (2013). Do high concentrations of microcystin prevent *Daphnia* control of phytoplankton? Water Research, 47(6), 1961–1970. https://doi.org/10.1016/j.watres.2012.12.038 23395484

[CIT0015] Chorus, I., & Welker, M. (2021). Toxic cyanobacteria. In Water: A guide to their public health consequences, monitoring and management (2nd ed.) CRC Press.

[CIT0016] Crain, C. M., Kroeker, K., & Halpern, B. S. (2008). Interactive and cumulative effects of multiple human stressors in marine systems. Ecology Letters, 11(12), 1304–1315. https://doi.org/10.1111/j.1461-0248.2008.01253.x 19046359

[CIT0017] DeWitt, T. J., & Scheiner, S. M. (2004). Phenotypic plasticity: Functional and conceptual approaches. Oxford University Press.

[CIT0018] Dowen, R. H., & Ahmed, S. (2019). Maternal inheritance: Longevity programs nourish progeny via yolk. Current Biology, 29(15), R748–R751. https://doi.org/10.1016/j.cub.2019.06.050 31386852

[CIT0019] Evans, M. L., Wilke, N. F., O’Reilly, P. T., & Fleming, I. A. (2014). Transgenerational effects of parental rearing environment influence the survivorship of captive-born offspring in the wild. Conservation Letters, 7, 371–379.

[CIT0020] Feiner, N., Radersma, R., Vasquez, L., Ringnér, M., Nystedt, B., Raine, A., Tobi, E. W., Heijmans, B. T., & Uller, T. (2022). Environmentally induced DNA methylation is inherited across generations in an aquatic keystone species. iScience, 25(5), 104303. https://doi.org/10.1016/j.isci.2022.104303 35573201 PMC9097707

[CIT0021] Felsenfeld, G. (2014). A brief history of epigenetics. Cold Spring Harbor Perspectives in Biology, 6(1), a018200. https://doi.org/10.1101/cshperspect.a018200 24384572 PMC3941222

[CIT0022] Fitz-James, M. H., & Cavalli, G. (2022). Molecular mechanisms of transgenerational epigenetic inheritance. Nature Reviews Genetics, 23(6), 325–341. https://doi.org/10.1038/s41576-021-00438-5 PMC7619059 34983971

[CIT0023] Fox, R. J., Donelson, J. M., Schunter, C., Ravasi, T., & Gaitán-Espitia, J. D. (2019). Beyond buying time: The role of plasticity in phenotypic adaptation to rapid environmental change. Philosophical Transactions of the Royal Society of London, Series B: Biological Sciences, 374(1768), 20180174. https://doi.org/10.1098/rstb.2018.0174 30966962 PMC6365870

[CIT0024] Fusco, G., & Minelli, A. (2010). Phenotypic plasticity in development and evolution: Facts and concepts. Philosophical Transactions of the Royal Society of London, Series B: Biological Sciences, 365(1540), 547–556. https://doi.org/10.1098/rstb.2009.0267 20083631 PMC2817147

[CIT0025] Geoghegan, J. L., & Spencer, H. G. (2013). Exploring epiallele stability in a population-epigenetic model. Theoretical Population Biology, 83, 136–144. https://doi.org/10.1016/j.tpb.2012.09.001 23044385

[CIT0026] Ger, K. A., Urrutia-Cordero, P., Frost, P. C., Hansson, L.-A., Sarnelle, O., Wilson, A. E., & Lürling, M. (2016). The interaction between cyanobacteria and zooplankton in a more eutrophic world. Harmful Algae, 54, 128–144. https://doi.org/10.1016/j.hal.2015.12.005 28073472

[CIT0027] Ghalambor, C. K., McKay, J. K., Carroll, S. P., & Reznick, D. N. (2007). Adaptive versus non-adaptive phenotypic plasticity and the potential for contemporary adaptation in new environments. Functional Ecology, 21(3), 394–407. https://doi.org/10.1111/j.1365-2435.2007.01283.x

[CIT0028] Gillis, M. K., & Walsh, M. R. (2019). Individual variation in plasticity dulls transgenerational responses to stress. Functional Ecology, 33(10), 1993–2002. https://doi.org/10.1111/1365-2435.13409

[CIT0029] Guerrero-Bosagna, C. (2017). Evolution with no reason: A neutral view on epigenetic changes, genomic variability, and evolutionary novelty. Bioscience, 67(5), 469–476. https://doi.org/10.1093/biosci/bix021

[CIT0030] Hairston, N. G. Jr, Holtmeier, C. L., Lampert, W., Weider, L. J., Post, D. M., Fischer, J. M., Cáceres, C. E., Fox, J. A., & Gaedke, U. (2001). Natural selection for grazer resistance to toxic cyanobacteria: Evolution of phenotypic plasticity? Evolution, 55(11), 2203–2214. https://doi.org/10.1111/j.0014-3820.2001.tb00736.x 11794781

[CIT0031] Hairston, N. G. Jr, Lampert, W., Cáceres, C. E., Holtmeier, C. L., Weider, L. J., Gaedke, U., Fischer, J. M., Fox, J. A., & Post, D. M. (1999). Lake ecosystems: Rapid evolution revealed by dormant eggs. Nature, 401(6752), 446–446. https://doi.org/10.1038/46731

[CIT0032] Harke, M. J., Steffen, M. M., Gobler, C. J., Otten, T. G., Wilhelm, S. W., Wood, S. A., & Paerl, H. W. (2016). A review of the global ecology, genomics, and biogeography of the toxic cyanobacterium, *Microcystis* spp. Harmful Algae, 54, 4–20. https://doi.org/10.1016/j.hal.2015.12.007 28073480

[CIT0033] Harmon, E. A., & Pfennig, D. W. (2021). Evolutionary rescue via transgenerational plasticity: Evidence and implications for conservation. Evolution and Development, 23(4), 292–307. https://doi.org/10.1111/ede.12373 33522673

[CIT0034] Harris, K. D. M., Bartlett, N. J., & Lloyd, V. K. (2012). *Daphnia* as an emerging epigenetic model organism. Genetics Research International, 2012, 1–8. https://doi.org/10.1155/2012/147892 PMC3335723 22567376

[CIT0035] Harrisson, K. A., Pavlova, A., Telonis-Scott, M., & Sunnucks, P. (2014). Using genomics to characterize evolutionary potential for conservation of wild populations. Evolutionary Applications, 7, 1008–1025. 25553064 10.1111/eva.12149 PMC4231592

[CIT0036] Heard, E., & Martienssen, R. A. (2014). Transgenerational epigenetic inheritance: Myths and mechanisms. Cell, 157(1), 95–109. https://doi.org/10.1016/j.cell.2014.02.045 24679529 PMC4020004

[CIT0037] Hendry, A. P., & Kinnison, M. T. (1999). Perspective: The pace of modern life: Measuring rates of contemporary microevolution. Evolution, 53(6), 1637–1653. https://doi.org/10.1111/j.1558-5646.1999.tb04550.x 28565449

[CIT0038] Herman, J. J., Spencer, H. G., Donohue, K., & Sultan, S. E. (2014). How stable “SHOULD” epigenetic modifications be? Insights from adaptive plasticity and bet hedging. Evolution, 68(3), 632–643. https://doi.org/10.1111/evo.12324. 24274594

[CIT0039] Hoffmann, A. A., & Sgrò, C. M. (2011). Climate change and evolutionary adaptation. Nature, 470(7335), 479–485. https://doi.org/10.1038/nature09670 21350480

[CIT0040] Houri-Zeevi, L., & Rechavi, O. (2017). A matter of time: Small RNAs regulate the duration of epigenetic inheritance. Trends in Genetics, 33(1), 46–57. https://doi.org/10.1016/j.tig.2016.11.001 27939252

[CIT0041] Isanta-Navarro, J., Hairston, N. G. Jr, Beninde, J., Meyer, A., Straile, D., Möst, M., & Martin-Creuzburg, D. (2021). Reversed evolution of grazer resistance to cyanobacteria. Nature Communications, 12(1), 1945. https://doi.org/10.1038/s41467-021-22226-9 PMC8007715 33782425

[CIT0042] Jablonka, E. (2017). The evolutionary implications of epigenetic inheritance. Interface Focus, 7(5), 20160135. https://doi.org/10.1098/rsfs.2016.0135 28839916 PMC5566804

[CIT0043] Jablonka, E., & Lamb, M. J. (2010). Transgenerational epigenetic inheritance. In Pigliucci, M., & Müller, G. B. (Eds.), *Evolution – the Extended Synthesis* (pp. 137–74). MIT Press.

[CIT0044] Jablonka, E., & Raz, G. (2009). Transgenerational epigenetic inheritance: Prevalence, mechanisms, and implications for the study of heredity and evolution. Quarterly Review of Biology, 84(2), 131–176. https://doi.org/10.1086/598822 19606595

[CIT0045] Jirtle, R. L., & Skinner, M. K. (2007). Environmental epigenomics and disease susceptibility. Nature Reviews Genetics, 8(4), 253–262. https://doi.org/10.1038/nrg2045 PMC5940010 17363974

[CIT0046] Kilham, S. S., Kreeger, D. A., Lynn, S. G., Goulden, C. E., & Herrera, L. (1998). COMBO: A defined freshwater culture medium for algae and zooplankton. Hydrobiologia, 377, 147–159.

[CIT0047] Kronholm, I., & Collins, S. (2016). Epigenetic mutations can both help and hinder adaptive evolution. Molecular Ecology, 25(8), 1856–1868. https://doi.org/10.1111/mec.13296 26139359

[CIT0048] Lacal, I., & Ventura, R. (2018). Epigenetic inheritance: Concepts, mechanisms and perspectives. Frontiers in Molecular Neuroscience, 11, 292. https://doi.org/10.3389/fnmol.2018.00292 30323739 PMC6172332

[CIT0049] Lachmann, M., & Jablonka, E. (1996). The inheritance of phenotypes: An adaptation to fluctuating environments. Journal of Theoretical Biology, 181(1), 1–9. https://doi.org/10.1006/jtbi.1996.0109 8796186

[CIT0050] Lavergne, S., Mouquet, N., Thuiller, W., & Ronce, O. (2010). Biodiversity and climate change: Integrating evolutionary and ecological responses of species and communities. Annual Review of Ecology Evolution, 41(1), 321–350. https://doi.org/10.1146/annurev-ecolsys-102209-144628

[CIT0051] Lind, M. I., & Spagopoulou, F. (2018). Evolutionary consequences of epigenetic inheritance. Heredity, 121(3), 205–209. https://doi.org/10.1038/s41437-018-0113-y 29976958 PMC6082883

[CIT0052] Lockwood, B. L., Julick, C. R., & Montooth, K. L. (2017). Maternal loading of a small heat shock protein increases embryo thermal tolerance in *Drosophila melanogaster*. Journal of Experimental Biology, 220(Pt 23), 4492–4501. https://doi.org/10.1242/jeb.164848 29097593 PMC5769566

[CIT0053] Merilä, J., & Hendry, A. P. (2014). Climate change, adaptation, and phenotypic plasticity: The problem and the evidence. Evolutionary Applications, 7(1), 1–14. https://doi.org/10.1111/eva.12137 24454544 PMC3894893

[CIT0054] Murray, K. O., Clanton, T. L., & Horowitz, M. (2022). Epigenetic responses to heat: From adaptation to maladaptation. Experimental Physiology, 107(10), 1144–1158. https://doi.org/10.1113/EP090143 35413138 PMC9529784

[CIT0055] Nilsson, E. E., McBirney, M., De Santos, S., King, S. E., Beck, D., Greeley, C., Holder, L. B., & Skinner, M. K. (2023). Multiple generation distinct toxicant exposures induce epigenetic transgenerational inheritance of enhanced pathology and obesity. Environmental Epigenetics, 9(1), dvad006. https://doi.org/10.1093/eep/dvad006 38162685 PMC10756336

[CIT0056] O’Dea, R. E., Noble, D. W. A., Johnson, S. L., Hesselson, D., & Nakagawa, S. (2016). The role of non-genetic inheritance in evolutionary rescue: Epigenetic buffering, heritable bet hedging and epigenetic traps. Environmental Epigenetics, 2, dvv014. 29492283 10.1093/eep/dvv014 PMC5804513

[CIT0057] OECD (2012). Test No. 211: Daphnia magna reproduction test, OECD guidelines for the testing of chemicals, section 2. OECD Publishing.

[CIT0058] Orsini, L., Spanier, K. I., & DE Meester, L. (2012). Genomic signature of natural and anthropogenic stress in wild populations of the waterflea *Daphnia magna*: Validation in space, time and experimental evolution. Molecular Ecology, 21, 2160–2175. 22257313 10.1111/j.1365-294X.2011.05429.x

[CIT0059] Pigliucci, M. (2001). Phenotypic plasticity: Beyond nature and nurture. JHU Press.

[CIT0060] Plaistow, S. J., Lapsley, C. T., & Benton, T. G. (2006). Context-dependent intergenerational effects: The interaction between past and present environments and its effect on population dynamics. American Naturalist, 167(2), 206–215. https://doi.org/10.1086/499380 16670981

[CIT0061] Proulx, S. R., Dey, S., Guzella, T., & Teotónio, H. (2019). How differing modes of non-genetic inheritance affect population viability in fluctuating environments. Ecology Letters, 22, 1767–1775. 31436016 10.1111/ele.13355

[CIT0062] Proulx, S. R., & Teotónio, H. (2017). What kind of maternal effects can be selected for in fluctuating environments? American Naturalist, 189(6), E118–E137. https://doi.org/10.1086/691423 28514627

[CIT0064] R Core Team. (2022). *R: A language and environment for statistical computing*.

[CIT0063] Rando, O. J., & Verstrepen, K. J. (2007). Timescales of genetic and epigenetic inheritance. Cell, 128(4), 655–668. https://doi.org/10.1016/j.cell.2007.01.023 17320504

[CIT0065] Rennison, D. J., Rudman, S. M., & Schluter, D. (2019). Genetics of adaptation: Experimental test of a biotic mechanism driving divergence in traits and genes. Evolution Letters, 3(5), 513–520. https://doi.org/10.1002/evl3.135 31636943 PMC6791182

[CIT0066] Rivoire, O., & Leibler, S. (2014). A model for the generation and transmission of variations in evolution. Proceedings of the National Academy of Sciences of the United States of America, 111(19), E1940–E1949. https://doi.org/10.1073/pnas.1323901111 24763688 PMC4024917

[CIT0067] Rodriguez-Cabal, M. A., Barrios-Garcia, M. N., Rudman, S. M., McKown, A. D., Sato, T., & Crutsinger, G. M. (2017). It is about time: Genetic variation in the timing of leaf-litter inputs influences aquatic ecosystems. Freshwater Biology, 62, 356–365.

[CIT0068] Rohrlack, T., Christoffersen, K., Dittmann, E., Nogueira, I., Vasconcelos, V., & Börner, T. (2005). Ingestion of microcystins by *Daphnia*: Intestinal uptake and toxic effects. Limnology and Oceanography, 50(2), 440–448. https://doi.org/10.4319/lo.2005.50.2.0440

[CIT0069] Rudman, S. M., Greenblum, S. I., Rajpurohit, S., Betancourt, N. J., Hanna, J., Tilk, S., Yokoyama, T., Petrov, D. A., & Schmidt, P. (2022). Direct observation of adaptive tracking on ecological time scales in *Drosophila*. Science, 375, eabj7484. 35298245 10.1126/science.abj7484 PMC10684103

[CIT0070] Sarnelle, O., & Wilson, A. E. (2005). Local adaptation of *Daphnia pulicariato* toxic cyanobacteria. Limnology and Oceanography, 50(5), 1565–1570. https://doi.org/10.4319/lo.2005.50.5.1565

[CIT0071] Sengupta, T., Kaletsky, R., & Murphy, C. T. (2023). The logic of transgenerational inheritance: Timescales of adaptation. Annual Review of Cell and Developmental Biology, 39(1), 45–65. https://doi.org/10.1146/annurev-cellbio-020923-114620 37339681

[CIT0072] Shahmohamadloo, R. S., Bhavsar, S. P., Ortiz Almirall, X., Marklevitz, S. A. C., Rudman, S. M., & Sibley, P. K. (2023a). Lake Erie fish safe to eat yet afflicted by algal hepatotoxins. Science of the Total Environment, 861, 160474. https://doi.org/10.1016/j.scitotenv.2022.160474 36481113

[CIT0073] Shahmohamadloo, R. S., Frenken, T., Rudman, S. M., van West, P., Ibelings, B. W., & Trainer, V. L. (2023b). Diseases and disorders in fish due to harmful algal blooms. In P. T. K. Woo, & R. P. Subasinghe (Eds.), Climate change on diseases and disorders of finfish in cage culture (3rd ed., pp. 387–429). CAB International.

[CIT0074] Shahmohamadloo, R. S., Ortiz Almirall, X., Holeton, C., Chong-Kit, R., Poirier, D. G., Bhavsar, S. P., & Sibley, P. K. (2019). An efficient and affordable laboratory method to produce and sustain high concentrations of microcystins by *Microcystis aeruginosa*. MethodsX, 6, 2521–2535. https://doi.org/10.1016/j.mex.2019.10.024 31763185 PMC6861626

[CIT0075] Shahmohamadloo, R. S., Ortiz Almirall, X., Simmons, D. B. D., Lumsden, J. S., Bhavsar, S. P., Watson-Leung, T., Eyken, A. V., Hankins, G., Hubbs, K., Konopelko, P., Sarnacki, M., Strong, D., & Sibley, P. K. (2021). Cyanotoxins within and outside of microcystis aeruginosa cause adverse effects in rainbow trout (*Oncorhynchus mykiss*). Environmental Science and Technology, 55(15), 10422–10431. https://doi.org/10.1021/acs.est.1c01501 34264629

[CIT0076] Shahmohamadloo, R. S., Poirier, D. G., Ortiz Almirall, X., Bhavsar, S. P., & Sibley, P. K. (2020a). Assessing the toxicity of cell-bound microcystins on freshwater pelagic and benthic invertebrates. Ecotoxicology and Environment Safety, 188, 109945. https://doi.org/10.1016/j.ecoenv.2019.109945 31753309

[CIT0077] Shahmohamadloo, R. S., Rudman, S. M., Clare, C. I., Westrick, J. A., Wang, X., De Meester, L., & Fryxell, J. M. (2024). Intraspecific genetic variation is critical to robust toxicological predictions in *Daphnia*. *Scientific Reports*, 14(1), 25883. 39468236 10.1038/s41598-024-76734-x PMC11519591

[CIT0078] Shahmohamadloo, R. S., Simmons, D. B. D., & Sibley, P. K. (2020b). Shotgun proteomics analysis reveals sub-lethal effects in *Daphnia magna* exposed to cell-bound microcystins produced by *Microcystis aeruginosa*. Comparative Biochemistry and Physiology Part D: Genomics and Proteomics, 33, 100656. https://doi.org/10.1016/j.cbd.2020.100656 32035333

[CIT0088] Shahmohamadloo, R. S., Gabidulin, A. R., Andrews, E. R., Fryxell, J. M., & Rudman, S. M. (2025). A test for microbiome-mediated rescue via host phenotypic plasticity in Daphnia. *Proceedings of the Royal Society B*. https://doi.org/10.1098/rspb.2025.0365 PMC11978444 40199359

[CIT0079] Skinner, M. K. (2015). Environmental epigenetics and a unified theory of the molecular aspects of evolution: A Neo-Lamarckian concept that facilitates Neo-Darwinian evolution. Genome Biology and Evolution, 7(5), 1296–1302. https://doi.org/10.1093/gbe/evv073. 25917417 PMC4453068

[CIT0080] Stajic, D., Bank, C., & Gordo, I. (2022). Adaptive potential of epigenetic switching during adaptation to fluctuating environments. Genome Biology and Evolution, 14(5), evac065. https://doi.org/10.1093/gbe/evac065 35567483 PMC9113428

[CIT0081] Uller, T. (2019). Chapter 15: Evolutionary perspectives on transgenerational epigenetics. In T. O. Tollefsbol, (Ed.), Transgenerational epigenetics (2nd ed., pp. 333–350). Academic Press.

[CIT0082] Urban, M. C., Bocedi, G., Hendry, A. P., Mihoub, J.-B., Pe’er, G., Singer, A., Bridle, J. R., Crozier, L. G., De Meester, L., Godsoe, W., Gonzalez, A., Hellmann, J. J., Holt, R. D., Huth, A., Johst, K., Krug, C. B., Leadley, P. W., Palmer, S. C. F., Pantel, J. H., & Travis, J. M. J. (2016). Improving the forecast for biodiversity under climate change. Science, 353(6304), aad8466. 27609898 10.1126/science.aad8466

[CIT0083] Vogt, G. (2021). Epigenetic variation in animal populations: Sources, extent, phenotypic implications, and ecological and evolutionary relevance. Journal of Bioscience, 46, 24. 33737497

[CIT0084] Walsh, M. R., & Gillis, M. K. (2021). Transgenerational plasticity in the eye size of *Daphnia*. Biology Letters, 17(6), 20210143. https://doi.org/10.1098/rsbl.2021.0143 34129799 PMC8205523

[CIT0085] Webster, A. K., & Phillips, P. C. (2024). Heritable epigenetic variation facilitates long-term maintenance of epigenetic and genetic variation. G3, 14(2), jkad287. https://doi.org/10.1093/g3journal/jkad287 38113034 PMC10849368

[CIT0086] West-Eberhard, M. J. (2003). Developmental plasticity and evolution. Oxford University Press.

[CIT0087] Yin, J., Zhou, M., Lin, Z., Li, Q. Q., & Zhang, Y.-Y. (2019). Transgenerational effects benefit offspring across diverse environments: A meta-analysis in plants and animals. Ecology Letters, 22(11), 1976–1986. https://doi.org/10.1111/ele.13373 31436014

