# Supplementary material for: Transgenerational epigenetic inheritance increases trait variation but is not adaptive
Source: Evolution. 2025 Mar 11;79(6):1033–43. doi: 10.1093/evolut/qpaf050 (PMC12167597; doi:10.1093/evolut/qpaf050)
Supplement: qpaf050_suppl_Supplementary_Figures_S1-S3_Tables_S1-S2 [file qpaf050_suppl_supplementary_figures_s1-s3_tables_s1-s2.pdf]

Supporting Information

Transgenerational epigenetic inheritance increases trait variation but is not adaptive

René S. Shahmohamadloo*[1], John M. Fryxell[2], Seth M. Rudman*[1]

[1] School of Biological Sciences, Washington State University, Vancouver, WA, United States
[2] Department of Integrative Biology, University of Guelph, Guelph, ON, Canada

***Corresponding authors:**
René S. Shahmohamadloo, School of Biological Sciences, Washington State University, 14204 NE Salmon Creek Ave, Vancouver, WA 98686, USA. Email: rene.shahmohamadloo@wsu.edu
Seth M. Rudman, School of Biological Sciences, Washington State University, 14204 NE Salmon Creek Ave, Vancouver, WA 98686, USA. Email: seth.rudman@wsu.edu

This file includes: Figure S1, Figure S2, Figure S3, Table S1, Table S2

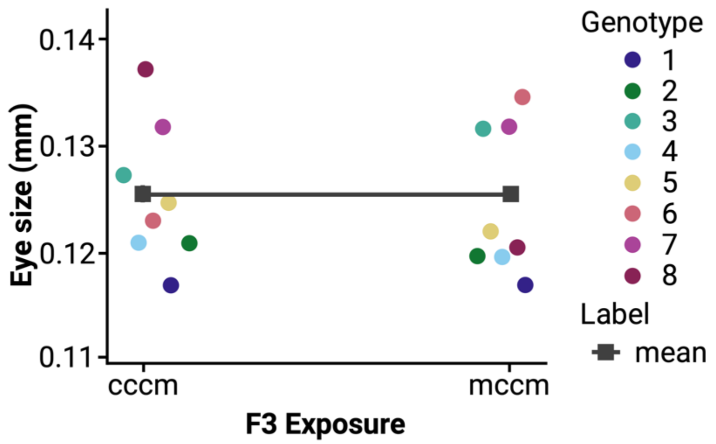

Figure S1. Phenotypic variation in eye size in *Daphnia magna* after four generations (P0 → F3) of transgenerational epigenetic inheritance. The mean (■) ±SE is also designated by a line. *D. magna* exposed to *Microcystis aeruginosa* in P0 and F3 are signified by 'mccm', and *D. magna* only exposed in F3 are signified by 'cccm'.

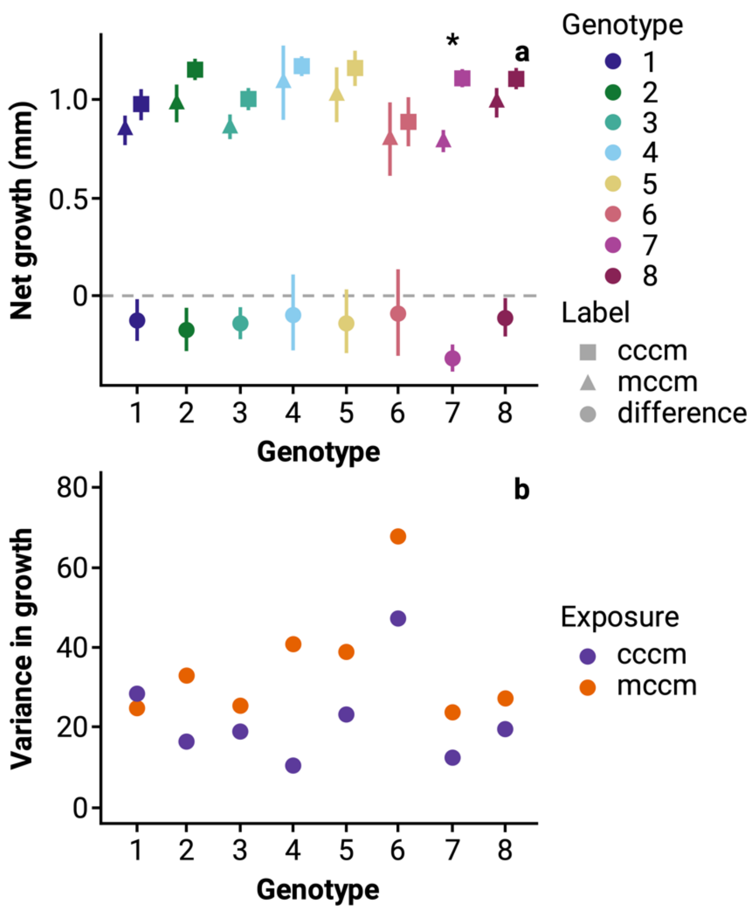

Figure S2. a) Difference in body growth ('mccm' - 'cccm') between exposures to *Microcystis aeruginosa* across each of eight *Daphnia magna* clonal populations in the F3 generation. The net body growth for 'cccm' ±SE (■), 'mccm' ±SE (▲), and the net difference between 'mccm' and 'cccm' ±SE (●) are listed for each genotype. Positive values (>0) indicate a beneficial effect on population dynamics, while negative values (<0) indicate a detrimental effect. Significance between treatments are designated by an asterisk (*) for each genotype. b) The variance in body growth of 'mccm' and 'cccm' exposures to *M. aeruginosa* across eight *D. magna* clones in the F3 generation.

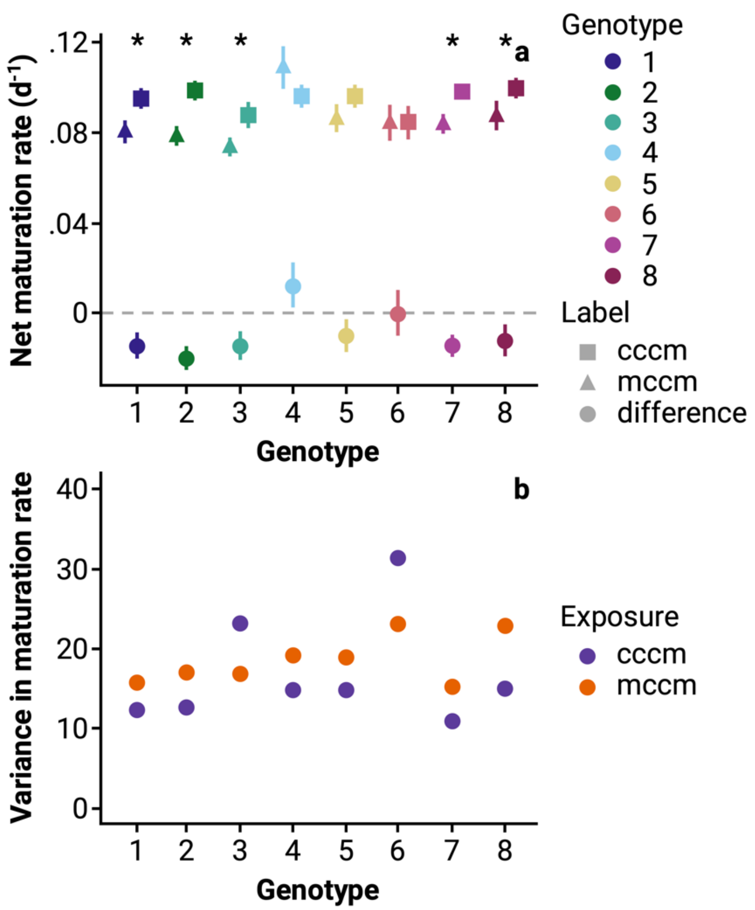

Figure S3. a) Difference in maturation rate (‘mccm’ - ‘cccm’) between exposures to *Microcystis aeruginosa* across each of eight *Daphnia magna* clonal populations in the F3 generation. The net maturation rate for ‘cccm’ ±SE (■), ‘mccm’ ±SE (▲), and the net difference between ‘mccm’ and ‘cccm’ ±SE (●) are listed for each genotype. Positive values (>0) indicate a beneficial effect on population dynamics, while negative values (<0) indicate a detrimental effect. Significance between treatments are designated by an asterisk (*) for each genotype. b) The variance in maturation rate of ‘mccm’ and ‘cccm’ exposures to *M. aeruginosa* across eight *D. magna* clones in the F3 generation.

Table S1. Statistical results for all measured traits (survival, body growth, neonate prod) from F3 exposure (‘mccm’ – ‘cccm’) and reaction norms from the difference in slopes between ‘cccc’-‘cccm’ (environmental effects only) and ‘mccc’-‘mccm’ (environmental + TEI effects) across the eight *Daphnia magna* clonal populations.

Trait	df (full, partial)	Test statistic	P-value
Survival			
F3 exposure	1,504	3.933	0.0001
Reaction norm	1,504	-2.291	0.0220
Growth			
F3 exposure	1,505	4.167	<0.0001
Reaction norm	1,504	-5.685	<0.0001
Neonate production			
F3 exposure	1,504	-1.623	0.1047
Reaction norm	1,504	-3.903	0.0001
Maturation rate			
F3 exposure	1,503	5.785	<0.0001
Reaction norm	1,502	-6.410	<0.0001
Eye size			
F3 exposure	1,505	-0.078	0.9380
Reaction norm	1,504	1.970	0.0494

Table S2. Statistical results for clonal differences in neonate production, body growth, and maturation rate between exposures to *Microcystis aeruginosa* ('mccm' – 'cccm') in the F3 generation.

Trait	Genotype	SE	df (full, partial)	Test statistic	P-value
Neonate production	1	0.248	1,188	1.338	0.1810
	2	0.212	1,188	-0.318	0.7505
	3	0.183	1,188	-1.965	0.0494
	4	0.325	1,188	0.324	0.7457
	5	0.253	1,188	-1.601	0.1093
	6	0.258	1,188	0.000	1.0000
	7	0.185	1,188	-2.667	0.0077
	8	0.199	1,188	1.277	0.2014
Growth	1	0.119	1,188	1.051	0.2946
	2	0104	1,188	1.648	0.1009
	3	0.0976	1,188	1.440	0.1514
	4	0.14	1,188	0.610	0.5428
	5	0.124	1,188	1.077	0.2830
	6	0.12	1,188	0.738	0.4615
	7	0.0995	1,188	3.185	0.0017
	8	0.103	1,188	1.084	0.2799
Maturation rate	1	0.00697	1,187	2.052	0.0416
	2	0.00604	1,187	3.297	0.0012
	3	0.00564	1,187	2.515	0.0128
	4	0.00809	1,187	-1.536	0.1263
	5	0.00716	1,187	1.394	0.1650
	6	0.00691	1,187	-0.004	0.9964
	7	0.00575	1,187	2.465	0.0146
	8	0.00594	1,187	2.025	0.0443
